# Evaluation of *Salvadora persica L.* and green tea anti-plaque effect: a randomized controlled crossover clinical trial

**DOI:** 10.1186/s12906-016-1487-0

**Published:** 2016-12-01

**Authors:** Hayder Raad Abdulbaqi, Wan Harun Himratul-Aznita, Nor Adinar Baharuddin

**Affiliations:** 1 Department of Restorative Dentistry, Faculty of Dentistry, University of Malaya, 50603 Kuala Lumpur, Malaysia; 2 Present address: Department of Periodontics, College of Dentistry, University of Baghdad, Bab-Almoadham, P.O. Box 1417, Baghdad, Iraq; 3 Department of Oral Craniofacial Sciences, Faculty of Dentistry, University of Malaya, 50603 Kuala Lumpur, Malaysia

**Keywords:** Salvadora, Tea, Dental plaque, Mouthwash, Clinical trial

## Abstract

**Background:**

In the author’s earlier in vitro investigation, a combination of 0.25 mg/ml green tea and 7.82 mg/ml *Salvadora persica L.* aqueous extracts was found to exhibit significant synergistic anti-bacterial and anti-adherence effects against primary plaque colonizers biofilm. A clinical trial was needed to support these preliminary in vitro results and to investigate its efficacy as a mouthwash in the control of dental plaque.

**Methods:**

A 24 h plaque re-growth, double-blinded, randomized crossover trial was carried out. Participants (n = 14) randomly rinsed with test formulation, 0.12% chlorhexidine (control) and placebo mouthwashes for 24 h. A week before the trial, all participants received scaling, polishing and oral hygiene education. On the trial day, the participants received polishing at baseline and rinsed with 15 ml of randomly allocated mouthwash twice daily without oral hygiene measures. After 24 h, plaque index was scored and then the participants entered a 6-days washout period with regular oral hygiene measures. The same protocol was repeated for the next 2 mouthwashes.

**Results:**

The results were expressed as mean (±SD) plaque index. The test mouthwash (0.931 ± 0.372) significantly reduced plaque accumulation when compared with placebo (1.440 ± 0.498, *p* < 0.0167) and chlorhexidine (1.317 ± 0.344, *p* < 0.0167) mouthwashes. No significant difference was found between chlorhexidine and placebo (*p >* 0.0167).

**Conclusions:**

The test mouthwash has an anti-plaque effect for a 24 h period. Longer-term clinical studies are highly encouraged to investigate its anti-plaque effect for longer periods.

**Trial registration:**

This study was registered in ClinicalTrials.gov as NCT02624336 in December 3, 2015.

**Electronic supplementary material:**

The online version of this article (doi:10.1186/s12906-016-1487-0) contains supplementary material, which is available to authorized users.

## Background

Dental plaque is the aetiology of caries and periodontal diseases. Thus, controlling dental plaque is crucial for oral health. Mechanical plaque control by using toothbrush and dental floss is the most adopted method of supragingival plaque control. Previous studies have reported low plaque removal performance using toothbrush, failure to use interproximal cleaning aids on a daily basis [1], and high prevalence of gingivitis among toothbrush users [2]. On the other hand, mechanical plaque control procedures merely focus on teeth, while gingivitis and periodontitis can develop from microbial plaque accumulated on oral soft tissue that serves as a source of bacteria to colonize tooth surface [3]. These findings suggest the need for using chemical plaque control to help in controlling dental plaque.

Chlorhexidine (CHX) is the gold standard mouthwash used for chemical plaque control [4]. Unfortunately, CHX has some undesirable effects such as extrinsic staining on teeth and restorations, interfering with taste function, bitter taste, enhancing calculus formation [5]. CHX mouthwashes containing anti-discolouration agents were reported to have no consistent beneficial effects on plaque and gingivitis [6]. This encourages many researchers to find alternatives for CHX.

Traditional medicinal plants may exhibit biological activities that enhance oral health. For example, *Salvadora persica L.,* family*: Salvadoraceae,* (Sp) root extracts is well documented for its anti-bacterial effect properties against dental plaque [7, 8]. It was reported that those using chewing sticks have saliva with significant lower level of primary plaque colonizers (including *Streptococcus mitis, Streptococcus sanguinis, Streptococcus oralis* and *Streptococcus salivarius)* as compared with those using conventional toothbrushes [9]. In addition to Sp, green tea (Gt), leafs of *Camellia sinensis var. assamica* (family*: Theaceae*), extracts have been reported to exhibit anti-bacterial effect against bacterial species of dental plaque [10, 11]. In an epidemiological study, Kushiyama et al. [12] concluded a modest inverse relationship between drinking one green tea cup/day and periodontal disease. Recently, brushing with dentifrice containing Gt was reported to enhance periodontal therapy outcome [13].

Primary plaque colonizers play an important initial role in the development of dental plaque [14]. In the author’s earlier in vitro investigation, a combination of 0.25 mg/ml Gt and 7.82 mg/ml Sp aqueous extracts was found to exhibit significant synergistic anti-bacterial and anti-adherence effects against primary plaque colonizers biofilm (*S. mitis*, *S. sanguinis*, and *Actinomyces viscosus*) [15]. In addition, a 24 h plaque re-growth protocol has been used successfully in earlier clinical studies to evaluate the anti-plaque efficacy of various oral formulations [16]. Thus, a 24 h plaque re-growth clinical trial was conducted to confirm these preliminary in vitro results and to evaluate its impact on plaque formation. Hence, this study hypothesized that the use of 0.25 mg/ml Gt and 7.82 mg/ml Sp aqueous extracts combination mouthwash would reduce plaque levels, when compared with a placebo, over a 24 h period.

## Methods

### Study design and population

This study was a 24 h plaque re-growth, double-blinded, randomized crossover clinical trial conducted at the Faculty of Dentistry, University of Malaya between February and June 2015. Participants who fulfilled the inclusion and exclusion criteria were invited to participate in this study. The inclusion criteria included participants (1) aged 25–40 years old; (2) in good general health and (3) with more than 20 teeth. The exclusion criteria included those presented with (1) active cavity caries and/or periodontal disease; (2) ongoing orthodontic treatment; (3) a history of antibiotics within the past 4 months (5) require prophylactic antibiotic coverage; (6) require systemic and/or topical non-steroidal anti-inflammatory drugs for the past 4 months; (7) pregnant or intended to and lactating mother; (8) known intolerance or allergy to mouthwashes; (9) have heart valve replacement and/or any systemic disease. In this study, written informed consents were obtained from all eligible participants prior to the beginning of the trial who passed through 3 weeks of preparatory (1 week) and trial (2 weeks) periods.

### Sample size

The sample size was calculated after conducting a pilot study on 5 participants. It was found that sample of 14 participants was enough to reject the null hypothesis between test and placebo at probability power of 0.95 and 0.05 type I error probability (see additional file 1).

### Clinical measurement

The plaque quantity was recorded using the modified Quigely Hein plaque index (PI) [17]. The data was recorded at labial/buccal and lingual/palatal surfaces of each disclosed tooth except wisdom teeth and any filled tooth surface. The distance from the gingival margin to the edge of the disclosed area was measured to the nearest 0.5 mm using a calibrated periodontal probe and the scores were recorded in a PI record form for each participant. The mean of PI for each participant was calculated by collecting the scores over the total number of surfaces examined. All plaque scores were recorded by single examiner. Alignment and assessment of the examiner were carried out with the assistance of highly experienced examiner as described by Hefti and Preshaw.[18] Absolute intra-examiner agreement, kappa value of 0.827, was achieved according to Landis and Koch [19] (see additional file 2).

### Intervention

The test formulation (patented, IP 2015704777), 0.12% chlorhexidine (control) and distilled water (placebo) were used in this study. A full description of the mouthwashes is illustrated in Table 1.

**Table 1:** Description of interventions

Interventions	CHX	Test (formulation)	Placebo
Ingredients & concentration	Chlorhexidine gluconate 0.12% (w/v) (active ingredient)	Combination of leafs of *Camellia sinensis var. assamica* (0.25 mg) + roots of *Salvadora persica L.* (7.82 mg) extracts/1 ml distilled water	Distilled water
Dosage/ Regimen	15 ml twice daily, rinse for 30 s, refrain from eating or drinking for 30 min	15 ml twice daily, rinse for 30 s, refrain from eating or drinking for 30 min	15 ml twice daily, rinse for 30 s, refrain from eating or drinking for 30 min
Duration	24 h	24 h	24 h
Color	Light blue	Light yellow	Colorless
Preparation	Used commercial oral rinse (Oradex™)	Prepared in Balai Ungku Aziz Research Laboratory (BUARL), Faculty of Dentistry University of Malaya	Prepared in Balai Ungku Aziz Research Laboratory (BUARL), Faculty of Dentistry University of Malaya

The mouthwashes were contained in identical opaque bottles which were randomly given sequential number codes (1, 2 and 3) by a laboratory technician not involved in this study. All participants had an equal probability of assignment to the interventions sequence which was randomly selected by the laboratory technician by using a computer random number generator (Microsoft Excel 2010). The examiner received a number-coded interventions sequence list to assign the blinded intervention.

To achieve allocation concealment, this trial was double-blinded in which examiner, nursing staff and participants were unable to identify the corresponding interventions contained in identical opaque bottles. Decoding was done after the end of the study.

### Plant extracts

Full information of leafs of *Camellia sinensis var. assamica* (family*: Theaceae*) and roots of *Salvadora persica L.* (family*: Salvadoraceae*) source, their aqueous extract preparation and their combination formulation were described in [15].

### Preparatory period

One week prior trial period, the aim and flow of the clinical trial were explained to the participants who were given oral hygiene education, and received professional scaling and polishing. All the participants were supplied with similar start-up pack containing fluoridated toothpastes, dental flosses and soft toothbrushes that they had to use during the preparatory phase before the clinical trial (7 days) and each wash out period (6 days).

### Clinical trial period

All participants’ teeth were disclosed with a disclosing agent by chewing an erythrosine tablet for 1 min and rinse their mouth with water 3 times, and then the participants’ teeth were polished to have plaque free tooth surfaces at the baseline. After that, the participants were asked to rinse with undiluted 15 ml of the allocated intervention for 30 s under supervision and instructed to prevent from eating and drinking for 30 min after rinsing. After the first rinsing, the participants were asked to rinse again at home after 12 h and to refrain from mechanical oral hygiene measures and using chewing gum for 24 h at which the teeth were again disclosed, PI was recorded and finally their teeth were polished. The empty opaque bottles were retrieved from the participants to ensure their compliance. Then, all participants entered a 6-days washout period and this protocol was repeated for the next 2 interventions. On the last day of the study, each participant received professional polishing of all his/her teeth.

### Safety

Oral examinations of oral surfaces including the buccal, labial and sublingual mucosa, gingiva, tongue, mucobuccal fold, hard and soft palate, uvula, oropharynx, teeth and dental restorations were carried out for all participants at the time of PI scoring after rinsing twice daily for 24 h following each intervention. All noticeable worsening adverse events were recorded except intervention-unrelated abnormalities, such as traumatic ulcers, food burns and lip bites.

### Statistical methods

Data analysis was done using Statistical Package of Social Science (SPSS) version 16.0. For interventions comparison, PI scores were described in term of mean and standard deviation. Kruskal-Wallis H test (*p* < 0.05) was used to detect any difference among interventions. Then, Mann-Whitney *U* test (*p* < 0.0167) was used to test the difference between each couple of interventions. The intra-examiner agreement was assessed by using kappa test. G*Power software (version 3.1.9.2) was used to determine sample size, intervention effect size and power of the study.

## Results

**Fig. 1:**
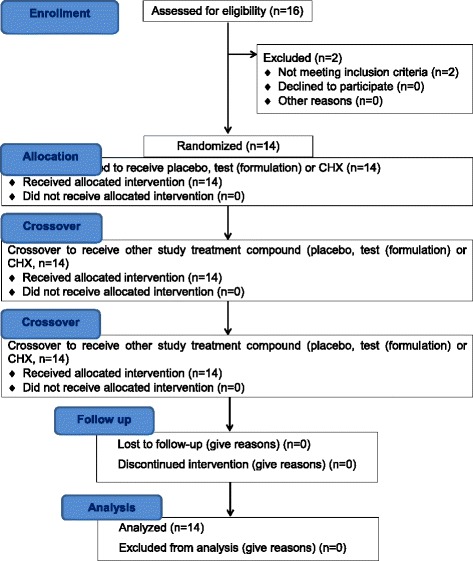
Consort 2010 flow diagram. The diagram graphically outlines the design and conduct of the clinical trial

A double-blind, randomized crossover trial was conducted to detect relative changes in plaque accumulation after 24 h re-growth by means of mean PI after rinsing with different interventions. The CONSORT 2010 flow diagram of this study is illustrated in Fig. 1. Out of the 16 subjects recruited at February 2015, only 14 subjects who fulfilled the study criteria began the trial and completed both preparatory and clinical trial periods. None of the participants was dropped out from the study. Demographic data of those participants **are** is shown in Table 2.

**Table 2:** Demographic data of participants

	Number	Total
		14
Age (years)	Mean ± SD	30.79 ± 5.22
	Range	25 – 39
Gender	Male	13
	Female	1
Ethnicity	Malay	5
	Others	9

All participants in this study rinsed with 15 ml of each intervention for 30 s. No noticeable harms were detected at the time of PI scoring after rinsing twice daily for 24 h. Mean PI showed significant difference between different groups at baseline i.e. 24 h after tooth polishing (*p* < 0.05). The test group (0.931 ± 0.372) had the lowest plaque scores followed by the CHX (1.317 ± 0.344) and placebo (1.440 ± 0.498) groups respectively as shown in Table 3.

**Table 3:** Mean and standard deviation of PI after treatment with the different interventions at 24 h after tooth polishing

Intervention	Number	Mean	SD	Kruskal-Wallis H test
Test (formulation)	14	0.931	±0.372	*p* < 0.05
CHX	14	1.317	±0.344	
Placebo	14	1.440	±0.498	

**Fig. 2:**
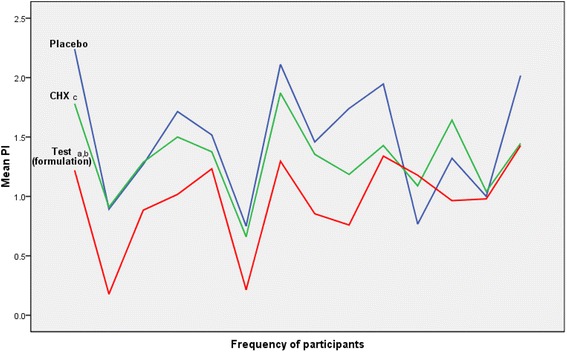
Mean PI of participants after treatment with different interventions at 24 h after tooth polishing. Statistical analysis of Mann Whitney *U* test showed (*a*) test (formulation) and placebo to be significant with *p* < 0.0167; (*b*) test (formulation) and CHX to be significant with *p* < 0.0167; (*c*) placebo and CHX to be non-significant with *p* >0.0167

The mean PI of the test group was significantly lower than mean PI of placebo (*p* < 0.0167) with an effect size of 1.158 and achieved power of 0.897 at α error probability 0.05. The interesting finding was that the mean PI of the test group was also significantly lower than that of the CHX group (*p* < 0.0167) with an effect size of 1.077 and power of 0.856. No significant difference between mean PI of CHX and placebo groups were found as shown in Table 4 and Fig. 2 (see additional file 1 and 3).

**Table 4:** Comparison between mean PI of different interventions groups with achieved effect size and power

Interventions groups comparison	Mann-Whitney *U* test	Achieved effect size	Achieved power at α error probability 0.05
Test (formulation) versus placebo	*p* < 0.0167	1.158	0.897
Test (formulation) versus CHX	*p* < 0.0167	1.077	0.856
CHX versus placebo	*p* > 0.0167	0.287	0.178

## Discussion

In this study, a combination of 0.25 mg/ml Gt and 7.82 mg/ml Sp aqueous extracts mouthwash (test) was able to significantly reduce plaque accumulation as compared to placebo after 24 h re-growth. Interestingly, this formulated mouthwash was also able to significantly reduce plaque quantity compared to the CHX mouthwash. In the authors’ earlier in vitro study, this same combination exhibited anti-bacterial activity comparable to 12% CHX against primary plaque colonizers, i.e. *S. mitis*, *S. sanguinis*, and *A. viscosus*. It also significantly reduced the adherence of those bacteria to saliva covered glass beads which acted as tooth surfaces. When compared the anti-adherence effect of the combination with CHX, fewer primary colonizers adhered but it was statistically non-significant [15].

The initial adherence of primary colonizers to oral surfaces is important as they provide new receptors for subsequent adhesion of secondary bacterial colonizers, thus promoting plaque development [14]. The finding suggested that the significant anti-plaque effect of the test combination was attributed, in addition to its anti-bacterial efficacy, to fewer primary colonizers’ receptors available on tooth surfaces for subsequent secondary colonizers adhesion, therefore resulting in retarded plaque development. This proposed explanation is further supported by earlier reported finding of lower salivary levels of primary colonizers among Sp chewing sticks users than toothbrush users; an effect attributed to chemical constituents of Sp such as thiocynate, sulfate, chloride, and nitrate [9]. Furthermore, polyphenolic tannins were reported to inhibit salivary α-amylase and bind both salivary histatin and proline-rich protein [20, 21]. Recently, it was reported that rinsing with a fluoride-based mouth rinse results in a slight fluoride accumulation at the surface of the pellicle-coated enamel [22]. Both tannins and fluoride were identified in Sp aqueous extracts [23] and they may bind salivary proteins of the pellicle covering tooth surfaces, modify the pellicle surface and therefore interfere with the adherence of primary plaque colonizers. On the other hand, gargling with Gt polyphenols, catechins, was reported to reduce intra-oral load of primary colonizers including *S. sanguinis*, *S. salivarius*, *S. mitis* and *Streptococcus sobrinus* [10]. Also, Gt was found to have an anti-adherence efficacy against oral streptococci [24]. These findings might be due to the capability of tea polyphenols to adsorb onto acquired pellicle, subsequently modify its structure, and thus interfere with the bacterial adherence to tooth surface [25].

In this study, the authors considered variations in participants such as gender, thus opted for a crossover design in which each participant would serve as his/her own control to improve the sensitivity for detection of relative changes in plaque accumulation. No mechanical hygiene measures for 24 h protocol was used because the deposition of dental plaque at the gingival margin occurs on all teeth surfaces which can be clinically recognized with or without disclosing agents in less than 24 h [26]. Also, 24 h plaque reformation can be safely measured as previously done [16]. Furthermore, in an earlier in vitro study the test mouthwash was found to exhibit significant anti-bacterial and anti-adherence activities [15]. Thus, the authors used the 24 h plaque re-growth protocol to confirm this in vitro result and to evaluate its impact on plaque formation using only the plaque index. Longer-term protocols, such as 4 days plaque re-growth design, were not been used in this study. This decision was made to avoid inappropriate evaluation of the anti-adherence effect of the test mouthwash, which is believed to be responsible for its anti-plaque effect. This is due to the fact that undisturbed plaque matures after 3 days of no oral hygiene measures [27]. However, the evaluation of the long-term effect of the test mouthwash on gingival tissue is highly recommended using a longer-term protocol and to include the gingival and bleeding indices. Absolute intra-examiner agreement, kappa value of 0.827, was achieved. This enhanced the reliability of the study in which the data was able to be measured in a reproducible manner which in turn improved the discriminative power of the study [18].

Earlier relevant clinical studies have investigated the anti-plaque efficacy of Gt and Sp extracts independently. It was reported that neither commercial Sp mouthwash (Persica^TM^) nor its comparator placebo reduced plaque accumulation [28]. In contrast, Al-Bayaty et al. [29] found that rinsing with 10 ml of Sp extract at 100 mg/ml thrice a day significantly reduced plaque scores but did not reach the better effect of CHX. Recent clinical trials reported that rinsing with Gt extracts at 50 and 250 mg/ml significantly reduced plaque accumulation and the anti-plaque effect of both concentrations was comparable to CHX [30, 31]. In conclusion, both Sp and Gt extracts have anti-plaque effect which is in accordance to the current results but at much higher concentrations. The novelty of this study relies on investigating the anti-plaque effect of the combination between Gt and Sp aqueous extracts at lower concentrations than previously studied. Thus, this has the advantage of both cost effect and safety.

In this study, rinsing with the test formulated mouthwash has significantly reduced plaque scores when compared to CHX with an effect size of 1.077. The comparison was powerful (achieved power = 0.856). This result was not surprising as the authors’ in vitro results had revealed better synergistic anti-adherence effect of the Gt and Sp extracts combination against primary colonizers than CHX, Gt extract and Sp extract alone. Less primary colonizers biofilm adhered to glass beads treated with the combination when compared to CHX after 2 h [15]. Although the difference was not significant, but this might have an effect on the plaque quantity formed after 24 h by providing less binding sites for the secondary plaque colonizers in vivo. On the other hand, less plaque scores were recorded after rinsing with CHX mouthwash compared to placebo with no significance difference. This may be due to small effect size of CHX achieved in this study as shown in Table 4. However, it may need higher sample size of participants to uncover statistical significant difference between CHX and placebo.

In this study, the test formulated mouthwash has significantly reduced supragingival plaque in healthy gingiva participants with no adverse effect detected. The succession of supragingival plaque reformation is similar for both healthy and periodontitis subjects [26]. This clinical finding suggests that the anti-plaque effect could also be seen in periodontitis subjects. Therefore, the test mouthwash can be used by healthy or periodontitis subjects.

## Conclusion

This study demonstrates that rinsing with 15 ml of 0.25 mg/ml Gt and 7.82 mg/ml Sp aqueous extracts combination twice daily can significantly reduce plaque accumulation after 24 h re-growth. This result supports the earlier reported anti-bacterial and anti-adherence effects of this combination which may explain its significant anti-plaque effect. The anti-plaque effect of this combination was significantly better than 0.12% CHX. So, this combination provides a natural alternative mouthwash to CHX for controlling plaque up to 24 h period. However, longer-term clinical studies are highly encouraged to confirm these results for longer-term, and to evaluate the effect of this combination on gingival tissue.

## Additional files

Additional file 1: Raw data of sample size, power and effect size of the study. (PDF 226 kb)
Additional file 2: Raw data and statistics of intra-examiner calibration. (PDF 127 kb)
Additional file 3: Raw data and statistics of means Pl of participants for placebo, test and chlorhexidine mouth rinses. (PDF 158 kb)

## Abbreviations

*A. viscosus*: *Actinomyces viscosus*
BUARL: Balai ungku aziz research laboratory
CHX: Chlorhexidine
Gt: Green tea, leafs of *Camellia sinensis var. assamica*
PI: Plaque index
PPP: Post graduated research grant
*S. mitis*: *Streptococcus mitis*
*S. salivarius*: *Streptococcus salivarius*
*S. sanguinis*: *Streptococcus sanguinis*
Sp: *Salvadora persica L*
SPSS: Statistical package of social science
