# Supplementary material for: Evaluation of Salvadora persica L. and green tea anti-plaque effect: a randomized controlled crossover clinical trial
Source: BMC Complement Altern Med. 2016 Dec 1;16:493. doi: 10.1186/s12906-016-1487-0 (PMC5131433; doi:10.1186/s12906-016-1487-0)
Supplement: Additional file 1: — Raw data of sample size, power and effect size of the study. (PDF 226 kb) [file 12906_2016_1487_MOESM1_ESM.pdf]

Additional file 1– Raw data of sample size, power and effect size of the study

Sample size calculation using G*Power software (version 3.1.9.2)

Raw data of the pilot study

Patient name	Mean plaque index		
	Placebo	CHX	Test
Mazin	2.24	1.78	1.22
Sadam	1.269	1.288	0.884
Zohaib	1.517	1.375	1.232
Nashwan	0.892	0.91	0.178
Tamem	1.714	1.5	1.017
Average	1.526	1.371	0.906
SD	0.503	0.318	0.432

[1] -- *Tuesday, October 13, 2015 -- 21:05:31*

t tests – Means: Wilcoxon-Mann-Whitney test (two groups)

**Options:**	A.R.E. method		
**Analysis:**	A priori: Compute required sample size		
**Input:**	Tail(s)	=	One
	Parent distribution	=	Normal
	Effect size d	=	1.3223961
	α err prob	=	0.05
	Power (1-β err prob)	=	0.95
	Allocation ratio N2/N1	=	1
**Output:**	Noncentrality parameter δ	=	3.4189777
	Critical t	=	1.7088367
	Df	=	24.7380304
	Sample size group 1	=	14
	Sample size group 2	=	14
	Total sample size	=	28
	Actual power	=	0.9533660

power and effect size of the study calculation using G*Power software (version 3.1.9.2)

1- placebo & CHX
2- placebo & test
3- CHX & test

[1] -- Tuesday, October 13, 2015 -- 19:58:13

t tests - Means: Wilcoxon-Mann-Whitney test (two groups)

**Options:**	A.R.E. method		
**Analysis:**	Post hoc: Compute achieved power		
**Input:**	Tail(s)	=	One
	Parent distribution	=	Normal
	Effect size d	=	0.2873942
	α err prob	=	0.05
	Sample size group 1	=	14
	Sample size group 2	=	14
**Output:**	Noncentrality parameter δ	=	0.7430409
	Critical t	=	1.7088367
	Df	=	24.7380304
	Power (1-β err prob)	=	0.1782583

[2] -- Tuesday, October 13, 2015 -- 19:59:18

t tests - Means: Wilcoxon-Mann-Whitney test (two groups)

**Options:**	A.R.E. method		
**Analysis:**	Post hoc: Compute achieved power		
**Input:**	Tail(s)	=	One
	Parent distribution	=	Normal
	Effect size d	=	1.1580331
	α err prob	=	0.05
	Sample size group 1	=	14
	Sample size group 2	=	14
**Output:**	Noncentrality parameter δ	=	2.9940268
	Critical t	=	1.7088367
	Df	=	24.7380304
	Power (1-β err prob)	=	0.8971951

[3] -- Tuesday, October 13, 2015 -- 20:00:41

t tests - Means: Wilcoxon-Mann-Whitney test (two groups)

**Options:**	A.R.E. method		
**Analysis:**	Post hoc: Compute achieved power		
**Input:**	Tail(s)	=	One
	Parent distribution	=	Normal
	Effect size d	=	1.0773888
	α err prob	=	0.05
	Sample size group 1	=	14
	Sample size group 2	=	14
**Output:**	Noncentrality parameter δ	=	2.7855257
	Critical t	=	1.7088367
	Df	=	24.7380304
	Power (1-β err prob)	=	0.8561572
