# Supplementary material for: Evaluation of Salvadora persica L. and green tea anti-plaque effect: a randomized controlled crossover clinical trial
Source: BMC Complement Altern Med. 2016 Dec 1;16:493. doi: 10.1186/s12906-016-1487-0 (PMC5131433; doi:10.1186/s12906-016-1487-0)
Supplement: Additional file 2: — Raw data and statistics of intra-examiner calibration. (PDF 127 kb) [file 12906_2016_1487_MOESM2_ESM.pdf]

Additional file 2- Raw data and statistics of intra-examiner calibration

Plaque index scoring	
Hayder1	Hayder2
5	5
2	2
1	1
5	2
3	3
2	2
2	2
2	2
0	0
5	5
0	0
2	2
4	3
2	2
0	0
2	2
0	0
1	1
1	2
1	2
0	0
1	1
0	0
3	3
4	4
2	2
1	1
2	2
2	1
2	2
2	2
3	3
3	3
2	2
2	2
3	3
3	3
1	1
2	2
2	2
0	2
2	2
2	2
3	3
2	2
2	2
0	0
3	3

Statistics by Kappa test in SPSS

Case Processing Summary

	Cases					
	Valid		Missing		Total	
	N	Percent	N	Percent	N	Percent
Hayder1 * Hayder2	48	9.1%	478	90.9%	526	100.0%

Symmetric Measures

		Value	Asymp. Std. Error[a]	Approx. T[b]	Approx. Sig.
Measure of Agreement	Kappa	.827	.066	10.670	.000
N of Valid Cases		48			

a. Not assuming the null hypothesis.

b. Using the asymptotic standard error assuming the null hypothesis.
