# Supplementary material for: Evaluation of Salvadora persica L. and green tea anti-plaque effect: a randomized controlled crossover clinical trial
Source: BMC Complement Altern Med. 2016 Dec 1;16:493. doi: 10.1186/s12906-016-1487-0 (PMC5131433; doi:10.1186/s12906-016-1487-0)
Supplement: Additional file 3: — Raw data and statistics of means Pl of participants for placebo, test and chlorhexidine mouth rinses. (PDF 158 kb) [file 12906_2016_1487_MOESM3_ESM.pdf]

Additional file 3- Raw data and statistics of means Pl of participants for placebo (H2O), test and chlorhexidine (CHX) mouth rinses

Raw data

Patient	mouth rinse	mean PI
1	placebo	2.24
1	CHX	1.78
1	Test	1.22
2	placebo	0.892
2	CHX	0.91
2	Test	0.178
3	placebo	1.269
3	CHX	1.288
3	Test	0.884
4	placebo	1.714
4	CHX	1.5
4	Test	1.017
5	placebo	1.517
5	CHX	1.375
5	Test	1.232
6	placebo	0.75
6	CHX	0.66
6	Test	0.214
7	placebo	2.111
7	CHX	1.87
7	Test	1.296
8	placebo	1.458
8	CHX	1.354
8	Test	0.854
9	placebo	1.74
9	CHX	1.185
9	Test	0.759
10	placebo	1.946
10	CHX	1.428
10	Test	1.339
11	placebo	0.767
11	CHX	1.089
11	Test	1.178
12	placebo	1.321
12	CHX	1.642
12	Test	0.964
13	placebo	1
13	CHX	1.041
13	Test	0.979
14	placebo	2.017
14	CHX	1.446
14	Test	1.428

Statistics of means Pl of participants for test and CHX

Ranks

	mouthwash	N	Mean Rank	Sum of Ranks
Mean PI	CHX	14	18.39	257.50
	Test	14	10.61	148.50
	Total	28		

Test Statistics[b]

	Mean PI
Mann-Whitney U	43.500
Wilcoxon W	148.500
Z	-2.504
Asymp. Sig. (2-tailed)	.012
Exact Sig. [2*(1-tailed Sig.)]	.011[a]

a. Not corrected for ties.
b. Grouping Variable: mouthwash

Statistics of means Pl of participants for placebo and CHX

Ranks

	mouthwash	N	Mean Rank	Sum of Ranks
Mean PI	H2O	14	15.86	222.00
	CHX	14	13.14	184.00
	Total	28		

Test Statistics[b]

	Mean PI
Mann-Whitney U	79.000
Wilcoxon W	184.000
Z	-.873
Asymp. Sig. (2-tailed)	.383
Exact Sig. [2*(1-tailed Sig.)]	.401[a]

a. Not corrected for ties.

b. Grouping Variable: mouthwash

Statistics of means Pl of participants for placebo and test

Ranks

	mouthwash	N	Mean Rank	Sum of Ranks
Mean PI	H2O	14	18.36	257.00
	Test	14	10.64	149.00
	Total	28		

Test Statistics[b]

	Mean PI
Mann-Whitney U	44.000
Wilcoxon W	149.000
Z	-2.481
Asymp. Sig. (2-tailed)	.013
Exact Sig. [2*(1-tailed Sig.)]	.012[a]

a. Not corrected for ties.

b. Grouping Variable: mouthwash
